# Current management of massive hemorrhage in trauma

**DOI:** 10.1186/1757-7241-20-47

**Published:** 2012-07-09

**Authors:** Pär I Johansson, Jakob Stensballe, Sisse R Ostrowski

**Affiliations:** 1 Section for Transfusion Medicine, Capital Region Blood Bank, Rigshospitalet University of Copenhagen, Blegdamsvej 9, DK-2100, Copenhagen, Denmark; 2 Department of Anesthesiology, HOC, Rigshospitalet, University of Copenhagen, Blegdamsvej 9, DK-2100, Copenhagen, Denmark; 3 Department of Surgery, Center for Translational Injury Research (CeTIR),, University of Texas Medical School at Houston, Houston, TX, USA

**Keywords:** Massive transfusion, trauma, hemorrhage, TEG, coagulopathy, FFP, RBC, platelets, rFVIIa, fibrinogen, PCC, antifibrinolytics

## Abstract

Hemorrhage remains a major cause of potentially preventable deaths. Trauma and massive transfusion are associated with coagulopathy secondary to tissue injury, hypoperfusion, dilution, and consumption of clotting factors and platelets. Concepts of damage control surgery have evolved prioritizing early control of the cause of bleeding by non-definitive means, while hemostatic control resuscitation seeks early control of coagulopathy.

Hemostatic resuscitation provides transfusions with plasma and platelets in addition to red blood cells in an immediate and sustained manner as part of the transfusion protocol for massively bleeding patients. Although early and effective reversal of coagulopathy is documented, the most effective means of preventing coagulopathy of massive transfusion remains debated and randomized controlled studies are lacking.

Viscoelastical whole blood assays, like TEG and ROTEM however appear advantageous for identifying coagulopathy in patients with severe hemorrhage as opposed the conventional coagulation assays.

In our view, patients with uncontrolled bleeding, regardless of it´s cause, should be treated with hemostatic control resuscitation involving early administration of plasma and platelets and earliest possible goal-directed, based on the results of TEG/ROTEM analysis. The aim of the goal-directed therapy should be to maintain a normal hemostatic competence until surgical hemostasis is achieved, as this appears to be associated with reduced mortality.

## Introduction

Hemorrhage requiring massive transfusion remains a major cause of potentially preventable deaths. Trauma and massive transfusion are associated with coagulopathy secondary to tissue injury, hypoperfusion, dilution and consumption of clotting factors and platelets and coagulopathy, together with hypothermia and acidosis, forms a “lethal” triad [1]. Also, in the last 10–15 years there has been some paradigm shift regarding optimal resuscitation of bleeding trauma patients before definitive hemorrhage control is achieved. Aggressive fluid resuscitation increases blood pressure, reverses vasoconstriction, dislodges early formed thrombus, causes dilutional coagulopathy and metabolic acidosis and increases blood loss in experimental studies [2]. Accordingly previous guidelines [3] recommending that fresh frozen plasma (FFP) and platelets (PLT) should be administered only when a whole blood volume or more has been substituted and then according to conventional coagulation analyses is now considered obsolete since this strategy leads to dilutional coagulopathy and compromises hemostatic competence in the most severely bleeding patients [1]. Instead, limiting fluid resuscitation and applying the concept of permissive hypotension with the goal of achieving a palpable radialis pulse in patients has been advocated, whereas in patients with head injury a systolic blood pressure above 110 mmHg is recommended [4-7].

The current transfusion guidelines advocate the concept of hemostatic control resuscitation, i.e., supplementing large transfusions of red blood cells (RBC) with FFP and PLT to critically injured patients in an immediate and sustained manner is proposed [7-9]. The rationale for balanced administration of blood products is that it mimics the composition of circulating blood and, hence, transfusion of RBC, FFP and PLT in a unit-for-unit ratio is likely to both prevent and treat coagulopathy due to massive hemorrhage. This review describes the clinical problems associated with hemorrhage and massive transfusion in trauma.

## Coagulopathy in massive hemorrhage

### Dilution

The dilution of coagulation factors and platelets is an important cause of coagulopathy in massively transfused trauma patients [10]. The Advanced Trauma Life Support guideline recommends aggressive crystalloid resuscitation but the dilutional effects of such administration on coagulation competence are well described [11,12] and this strategy provokes acidosis, formation of interstitial oedema with tissue swelling, impairment of the microcirculation and hence compromised oxygenation [13,14].

Furthermore, synthetic colloid resuscitation fluids influence coagulation competence more profoundly than crystalloids. Hydroxyethyl starch (HES) causes efflux of plasma proteins from blood to the interstitial space, reduction in plasma concentration of coagulation factor VIII and von Willebrand factor (vWF), inhibition of platelet function and reduced interaction of activated FXIII with fibrin polymers [11,12,15].. This was further corroborated by a recent meta-analysis of 24 studies evaluating the safety of HES 130/0.4 administration in surgical, emergency and intensive care patients, with results demonstrating that HES administration promotes a dose-dependent coagulopathy [16]. Also, administration of blood products such as RBC, FFP and PLT may cause significant dilution since these blood products are stored in anticoagulation solutions reducing coagulation factor concentration to approximately 60% and platelet count to approximately 80x10^9^/l when a hematocrit of 30% is warranted [17].

### Hypothermia

Hypothermia is associated with risk of uncontrolled bleeding and death in trauma patients. Hypothermia induced coagulopathy is attributed to platelet dysfunction, reduced coagulation factor activity (significant below 33°C) [14,18], and induction of fibrinolysis [19] and these effects are reversible with normalization of body temperature.

### Acidosis

In trauma patients acidosis is often induced by hypoperfusion and excess administration of ionic chloride, i.e. NaCl during resuscitation [20]. Acidosis impairs almost all essential parts of the coagulation process: At pH < 7.4, platelets change their structure and shape [21]. The activity of coagulation factor complexes on the cell surface is reduced and the resulting impaired thrombin generation is a major cause of coagulopathic bleeding. Furthermore, acidosis leads to increased degradation of fibrinogen [22] which further aggravates the coagulopathy.

### Trauma

Brohi et al. [23-27] described an early “endogenous” coagulopathy in trauma patients not attributed to dilution and hypothermia with shock and hypoperfusion as the key drivers of acute traumatic coagulopathy through widespread activation of the anticoagulant and fibrinolytic pathways.

We recently suggested that the coagulopathy observed in trauma patients, which reflects the state of the fluid phase including its cellular elements i.e., circulating whole blood, is a consequence of the degree of the tissue injury and the thereby generated sympathoadrenal activity and importantly, critically related to the degree of endothelial damage, with a progressively more procoagulant endothelium (solid phase) inducing a gradient of increasing anticoagulation towards the fluid phase (circulating blood) [28]. Though it seems counterintuitive that increasing injury severity is associated with progressive hypocoagulability and hyperfibrinolysis [28,29], this may from an evolutionary perspective exert a survival advantage by preserving blood flow through the progressively more damaged and procoagulant microvasculature [28] In alignment with this, we found that in trauma patients upon hospital admission, a high level of syndecan-1, a marker of endothelial glycocalyx degradation, was associated with high sympathoadrenal activity and increased mortality, even after adjusting for injury severity score [30]. Also, only in patients with high syndecan-1 levels, was increasing injury severity associated with increased tissue and endothelial damage, protein C depletion, hyperfibrinolysis and inflammation [30].

### Consumptive coagulopathy

Tissue injury secondary to trauma induce immediate activation of the coagulation system through upregulation of tissue factor (TF) expression and extensive thrombin generation. Tissue injury in association with extensive endothelial injury, massive soft tissue damage, and fat embolization from long bone fractures, may be associated with consumption of coagulation factors and platelets and, hence development of coagulopathy. Disseminated intravascular coagulation (DIC) is the most extreme form of consumptive coagulopathy and is characterized by systemic activation of pathways leading to and regulating coagulation, which can result in the generation of fibrin clots that may cause organ failure with concomitant consumption of platelets and coagulation factors that may result in clinical bleeding [31,32]. We recently reported that disseminated intravascular coagulation (DIC) was not a part of the early coagulopathy secondary to trauma [33] though this may develop later in the course of resuscitation as described by Gando et al. [34].

### Hyperfibrinolysis

Increased fibrinolysis is observed in patients with profound endothelial activation and damage secondary to trauma, surgery and ischemia-reperfusion injury where tissue-type plasminogen activator (tPA) is released from the Weibel-Palade bodies of the endothelial cells. In trauma, increased fibrinolysis has been reported in the most severely injured patients and this correlates with poor outcome [29,35-38]. The presence of increased fibrinolysis with increasing injury severity probably reflects an evolutionary mechanism aiming at preventing fatal intravascular coagulation secondary to systemic hypercoagulation induced by the trauma, as reported recently by us [28].

## Anticoagulation

Vitamin K antagonists are frequently used by patients with atrial fibrillation or artificial cardiac valves and warfarin has been reported to be associated with about 21,000 visits for bleeding complications per year in the US alone [39], and these data are consistent with reports of major bleeding frequencies for warfarin as high as 10% to 16% [40]. The use of International Normalized Ratio (INR, a plasmatic coagulation analysis) to monitor the degree of anticoagulation by warfarin may in part explain this problem. The lack of adequate hemostatic monitoring is also evident with regards to the newer pharmaceutical agents used for secondary prevention and postoperative thromboprophylaxis such as the direct thrombin inhibitor dabigatran [41], the indirect FXa inhibitors apixaban [42] and rivaroxaban [43]. Furthermore, as for now it is recommended that treatment with these agents do not require hemostatic monitoring [44]. Despite this, however, reports of severe bleedings in patients taking these medications are being reported, including trauma,and since no antidote exists treatment of these patients is a major challenge [45].

Apart from coagulopathy due to iatrogenic heparinization, critically ill patients, including trauma patients, may become endogenously heparinized due to degradation of the endothelial glycocalyx [30,46], the antiadhesive and anticoagulant carbohydrate-rich surface layer that covers and protects the endothelial cells and contains significant amounts of heparin-like substances [47-53].

## Platelet inhibitors

An important cause of excessive bleeding in trauma patients is platelet inhibitors, which an increasing proportion of the population today uses as secondary prevention. Currently, the most important are the platelet ADP receptor inhibitors clopidogrel and lately also the even more potent prasugrel that irreversibly inhibits platelet activation through the platelet ADP receptor and confers more potent platelet inhibition than acetylsalicylic acid

Importantly, the enhanced antiplatelet activity and greater efficacy seen with prasugrel when compared to clopidogrel in clinical trials has been accompanied by increased bleeding risk and the FDA advisory committee issued guidance to physicians about increased risk in low-weight or elderly patients [54].

## Monitoring hemostasis

### Whole blood viscoelastical assays

**Figure 1:**
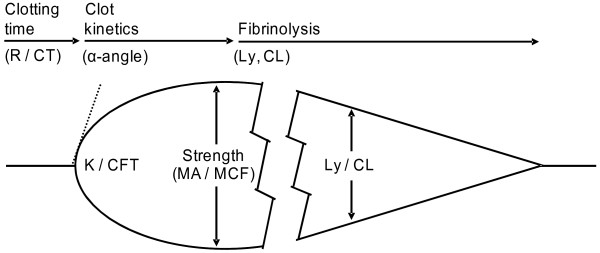
Schematic TEG/ROTEM trace indicating the commonly reported variables reaction time (R)/clotting time (CT), clot formation time (K, CFT), alpha angle (α), maximum amplitude (MA)/maximum clot firmness (MCF) and lysis (Ly)/clot lysis (CL).

**Figure 2:**
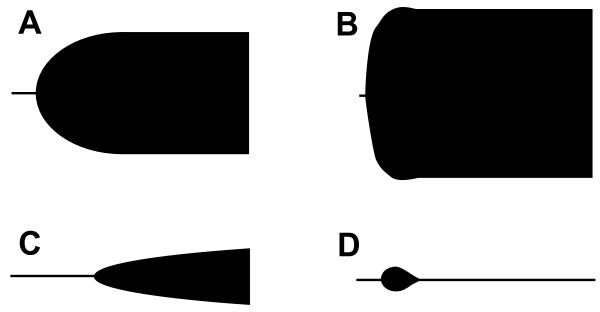
Schematic presentation of various VHA tracings: A) Normal, B) Hypercoagulability, C) Hypocoagulability (coagulation factor deficiency and thrombocytopenia/pathy and/or low fibrinogen) and D) Primary hyperfibrinolysis.

Introduction of the cell-based model of hemostasis emphasizes the role of platelets for intact thrombin generation and highlights the importance of the dynamics of thrombin generation influencing the quality and stability of the thrombus formed [55]. Consequently, hemostatic assays performed on plasma such as activated partial thromboplastin time (APTT) and prothrombin time (PT) are of limited value [56] and they do not correlate with clinically relevant coagulopathies [57,58]. Instead, employing a whole blood assay, such as viscoelastic hemostatic assays (VHA) like TEG/ROTEM that records the viscoelastic changes during clot formation and subsequent lysis is preferable. The TEG reports (Figure 1): R (reaction time), angle (α), the maximum amplitude (MA), the maximal clot strength; and clot lysis (Ly) [29,59-61]. Typical TEG profiles observed in trauma patients are normal, hypercoagulable, hypocoagulable and hyperfibrinolytic profiles (Figure 2), and in actively bleeding patients these are treated according to an algorithm (Table 1).

**Table 1:** Recommended TEG algorithm for goal-directed therapy of bleeding patients in the Capital Region of Denmark

TEG parameter*	**Coagulopathy**	**Intervention**
**R >10 min**	Coagulation factors ↓	FFP 10–20 ml/kg (if FFP is without clinical efficacy, consider cryoprecipitate 3–5 ml/kg)
**Angle <52 °**	Hypofibrinogenemia?	→ Functional Fibrinogen (FF) analysis
**MA <49 mm and**		
**MA**_**FF**_**<14 mm**	Fibrinogen ↓	FFP 20–30 ml/kg /
Fibrinogen konc. 25–50 mg/kg /		
Cryoprecipitate 5 ml/kg		
**MA <49 mm and**		
**MA**_**FF**_**>14 mm**	Platelets ↓	Platelets 5–10 ml/kg
**Ly30 >8%**	Primary hyperfibrinolysis	Tranexamic acid 1–2 g IV (adults)
Children 10–20 mg/kg IV		
**Ly30 >8% and**		
**Angle and/or MA ↑↑**	Reactive hyperfibrinolysis	Tranexamic acid contraindicated
**Difference in R >2 min between st-TEG and hep-TEG**	Heparinization	Protamine sulphate or FFP 20–30 ml/kg

R, Reaction time; Angle, α-angle; MA, Maximum amplitude; MA_FF_, Maximum amplitude by Functional Fibrinogen® analysis; Ly30, Lysis after 30 min; st-TEG, standard TEG; hep-TEG, heparinase TEG.

*Reference values (Haemonetics Corp.): R 3–8 min, Angle 55–78 °, MA 51–69 mm, Ly30 0-8%, MA_FF_=14-24 mm.

Reduced clot stability correlates with clinical bleeding conditions as demonstrated by Plotkin et al. [62] who, in patients with a penetrating trauma, reported TEG to be an accurate indicator of the blood product requirements. Furthermore, TEG is the gold standard for identifying hypercoagulability [63-65] and hyperfibrinolysis [35-38,65,66], the latter a significant cause of bleeding in patients with major trauma [29,35-38,59-61,66]. The use of VHA in trauma is now recommended by recent guidelines [67,68] and text books [69] and its use in the military field is extensive and many Level 1 trauma centers consider use of VHA to monitor and guide hemostatic therapy as routine.

### Whole blood platelet function analyzers

Different assays evaluating the degree of platelet inhibition secondary to platelet inhibitors exists. Light transmission aggregometry (LTA), previously considered the ”gold standard” for investigation of platelet aggregation [70], unfortunately relies on artificially manufactured platelet rich plasma suspensions, which do not reflect in vivo conditions and consequently platelet function assays performed on whole blood are today favored. Examples of whole blood platelet function assays are PFA100 (Siemens, Tarrytown, USA), VerifyNow (Accumetrics, San Diego, USA) and Multiplate (Verum Diagnostica GmbH, Munich, Germany), which all have been reported to be able to identify clinically relevant platelet inhibition secondary to pharmacological platelet inhibitors.

## Goal-directed hemostatic resuscitation based on VHA

Goal-directed treatment with blood products and antifibrinolytic pharmacological agents based on the result of the whole blood viscoelastic hemostatic assays (VHA), together with the clinical presentation, was introduced for more than 25 years ago in patients undergoing liver transplantation and cardiac surgery and there are validated algorithms for how coagulopathy is identified and treated in patients with ongoing bleeding, based on VHA [71]. More than 25 studies encompassing more than 4,500 patients have evaluated VHA vs. conventional coagulation assays on bleeding and transfusion requirements in patients undergoing cardiac, liver, vascular, or trauma surgery and in patients requiring massive transfusion. These studies demonstrate the superiority of VHA in predicting the need for blood transfusion, and the VHA-based algorithm reduces the transfusion requirements and the need for re-do surgery in contrast to treatment based on conventional coagulation assays [29] and this was further corroborated in a recent Cochrane review [72].

We found that implementation of goal-directed hemostatic resuscitation of massively bleeding patients, including trauma, based on the VHA reduced mortality by approximately 30% in patients requiring more than 10 RBC in the first 24 hours [71]. Systematic use of VHA to monitor and guide transfusion therapy is furthermore endorsed by several recent international transfusion guidelines and teaching books [68,69].

## Administration of blood products

### Red blood cells

In response to hemorrhage, lowered hematocrit contributes to coagulopathy since erythrocytes promote marginalization of platelets so the platelet concentrations along the endothelium remains almost seven times that of the average blood concentration [73]. In addition, erythrocytes support thrombin generation through exposure of procoagulant membrane phospholipids [1,74] and they activate platelets by liberating ADP [75,76] emphasizing that in vivo thrombus formation is a multicellular event [55,77]. Yet, the optimal hematocrit for platelet-vessel wall interactions remains unknown but it may be as high as 35% [78].

### Fresh frozen plasma

It remains controversial when and in what dose plasma should be transfused to massively bleeding trauma patients[79-87].

The optimal ratio of FFP to RBCs remains to be established although collectively the data indicate that a FFP:RBC ratio greater than 1:2 is associated with improved survival compared to one lower than 1:2 [79,80,83-85]. This is further supported by a review and meta-analysis from 2010 reporting that in patients undergoing massive transfusion, high FFP to RBC ratios was associated with a significant reduction in the risk of death (odds ratio (OR) 0.38 (95%CI 0.24-0.60) and multiorgan failure (OR 0.40 (95%CI 0.26-0.60) [88], and a meta-analysis from 2012 reports of reduced mortality in trauma patients treated with the highest FFP or PLT to RBC ratios [89].

### Platelets

Platelets are also pivotal for hemostasis [55,77] and several retrospective studies report an association between thrombocytopenia and postoperative bleeding and mortality [8,90,91]. Holcomb et al. [85] found that the highest survival was established in patients who received both a high PLT:RBC and a high FFP:RBC ratio. Inaba et al. recently reported from a retrospective study of massively transfused patients that as the apheresis platelet to RBC ratio increased, a stepwise improvement in survival was seen and a high apheresis PLT:RBC ratio was independently associated with improved survival [92]. This is in alignment with Brown et al. who reported that admission platelet count was inversely correlated with 24-hour mortality and transfusion of RBCs and that a normal platelet count may be insufficient after severe trauma suggesting these patients may benefit from a higher platelet transfusion threshold [93]. In a recent meta-analysis, a high PLT:RBC ratio in massively bleeding trauma patients was reported to reduce mortality [89].

### Massive transfusion protocols and ratios

A recent meta-analysis of retrospective observational studies evaluating the effect of FFP:RBC and/or PLT:RBC ratios and survival in massively bleeding trauma patients recently reported a significant survival benefit in patients receiving high FFP:RBC and PLT:RBC ratios [89]. A potential confounder of these results are survivorship bias relating to that those surviving long enough will receive FFP and PLT whereas those dying early will not, as reported by Snyder et al. [79]. This was recently corroborated by Ho et al. who employed mathematical modeling of the observational trauma studies performed involving FFP:RBC ratios concluding that some of these probably included survivorship bias in favor of a high FFP:RBC ratio [94].

Survivorship bias, however, does not explain the improved survival in studies concerning the introduction of transfusion packages where both FFP and PLT are immediately available i.e. where pre-thawed FFP are available. Cotton et al. [95] implemented a trauma exsanguination protocol (TEP) involving 10 RBC, 4 FFP and 2 apheresis PLT for trauma patients and used it to evaluate 211 trauma patients of who 94 received TEP and 117 were historic controls. The TEP patients received more RBC (16 vs. 11), FFP (8 vs. 4), and PLT (2 vs. 1) intraoperatively than the controls and displayed lower 30-day mortality (51% vs. 66%). After controlling for age, sex, mechanism of injury, Trauma and Injury Severity Score (TRISS), and 24-hour blood product usage, a 74% reduction in the odds ratio of mortality was found among patients in the TEP group. In a later study involving additionally 53 patients

Cotton also [96] reported that not only was 30-day survival higher in the TEP group compared to the controls, but the incidence of pneumonia, pulmonary failure and abdominal compartment syndrome was lower in the TEP patients. Also, the incidence of sepsis or septic shock and multi-organ failure was lower in TEP patients. Although the TEP group received more blood products intraoperatively, the 24 h transfusion requirements were lower than in controls, supporting that early and aggressive administration of plasma and platelets improves hemostasis and this is in alignment with the result of a recent meta-analysis of trauma patients requiring massive transfusion [89]. In alignment with this, Duchesne et al. reported that in trauma patients undergoing damage control laparotomy introduction of damage control resuscitation encompassing early administration of plasma and platelets together with RBC was associated with improved 30-day survival, (73.6% vs. 54.8%, p<0.009) when compared to patients treated with conventional resuscitation efforts [97]. Similarly, we evaluated 832 massively transfused patients, of whom 20% were trauma patients, two years prior toand two years after implementation of hemostatic control resuscitation in 2004 [71]. The concept involved pre-emptive use of PLT and FFP organized into transfusion packages (5 RBC, 5 pre-thawed FFP and 2 PLT; each a pool of 4 buffy coat platelets) to be administered to patients with uncontrollable bleeding. Compared to the controls, the intervention group received displayed a reduction in 30-day mortality (20% vs. 32% in controls, p=0.002).

A multicentre randomized control study evaluating the effect of different blood product ratios on survival in massively bleeding trauma patients will commence in the USA this year and hopefully this will result in evidence for how best to resuscitate these patients with blood products (http://www.uth.tmc.edu/cetir/PROPPR/index.html).

### Fresh whole blood

With the implementation of fractionated blood components, routine use of fresh whole blood (FWB) for resuscitation of bleeding patients was abandoned in the civilian setting. In the combat setting, however, FWB has been used in situations where fractionated blood products, and especially platelets were not available. In a report of US military patients in Iraq and Afghanistan from January 2004 to October 2007, those with hemorrhagic shock, a resuscitation strategy that included FWB was associated with improved 30-day survival (95% vs. 82%, p=0.002) [98] and an ongoing trial is currently addressing this issue (www.clingovtrial.com/ NCT01227005)..

It should be noted that administration of any blood product carries potential risks for the patient including viral and bacterial transmission, hemolytic transfusion reactions, transfusion related acute lung injury and immunomodulation and consequently transfusion of blood products should be reserved to patients who actually needs this therapy [99].

## Hemostatic agents

### Antifibrinolytics

Hyperfibrinolysis contribute significantly to coagulopathy and antifibrinolytics agents reduce the blood loss in patients with both normal and exaggerated fibrinolytic responses to surgery by preventing plasmin(ogen) from binding to fibrin and by preventing plasmin degradation of platelet glycoprotein Ib receptors [100,101]. In a placebo controlled randomized study (CRASH-2) including 20,211 adult trauma patients, tranexamic acid as compared to placebo significantly decreased all-cause mortality from 16.0% to 14.5%, p=0.0035 [102]. We recommend monitoring of hemostasis with TEG to identify hyperfibrinolytic states in trauma patients [35-38,65,66] and, consequently, targeted treatment with antifibrinolytic agents.

### Recombinant Factor VIIa

Recombinant factor VIIa (rFVIIa) acts in pharmacological doses by enhancing thrombin generation on the activated platelets independent of factor VIII and IX and is currently approved for episodes of severe hemorrhage or perioperative management of bleeding in patients with congenital factor VII deficiency and hemophilia A or B with inhibitors [103,104]. Data from the CONTROL trial, a phase 3 randomized clinical trial evaluating efficacy and safety of rFVIIa as an adjunct to direct hemostasis in major trauma [105], rFVIIa did not change mortality in patients with blunt (11.0% (rFVIIa) vs. 10.7% (placebo)) or penetrating (18.2% (rFVIIa) vs. 13.2% (placebo)) trauma. [106]In a recent review reporting on the rate of thromboembolic events in all published randomized placebo controlled trials of rFVIIa use [107]reported that the rates of arterial thromboembolic events among all subjects were higher among those who received rFVIIa than among those who received placebo (5.5% vs. 3.2%, p=0.003) [105].

### Fibrinogen concentrate

Conversion of sufficient amounts of fibrinogen to fibrin is a prerequisite for clot formation and reduction in the circulating level of fibrinogen due to consumption [32] and/or dilution by resuscitation fluids [108] induces coagulopathy [109]. Fibrinogen is the first hemostatic component that declines to pathologically low levels following trauma and/or hemodilution [108]. It is, therefore, important to maintain an adequate fibrinogen level when continued bleeding is bridged by saline and/or colloid infusion or blood products (primarily RBC) without fibrinogen. Recent data indicate that coagulopathy induced by synthetic colloids such as HES may be reversed by the administration of fibrinogen concentrate [110]. A recent review only found four studies of poor quality assessing fibrinogen concentrate to bleeding surgical and trauma patients and concluded that randomized controlled trials of sufficient size and long-term follow-up needs to be performed before such a practice can be recommended routinely [111]. The use of fibrinogen concentrate in patients with established hypofibrinogenemia, diagnosed by VHA (Functional Fibrinogen® or FibTEM®), in addition to a balanced administration of RBC, FFP and platelets, may however contribute to faster achievement of a normal hemostasis in massively bleeding patients, and we recommend the use of fibrinogen concentrate according to TEG guided algorithms in such patients (Table 1).

### Prothrombin complex concentrate

The four-factor PCC encompasses coagulation factors II, VII, IX and X that all are essential for thrombin generation. Administration of PCC is indicated for the treatment of congenital coagulation disorders and to reverse oral administered anticoagulation by vitamin K antagonists [112], whereas experience with treatment of massive bleeding with PCC is lacking. Recently, Carvalho et al. reported that administration of PCC to patients with massive bleedings had beneficial effect on hemostasis and this warrants further investigation in a randomized controlled setting [113].

### Factor XIII

Factor XIII is important for clot firmness by binding to platelets through the GPIIb/IIIa receptor and by cross-linking fibrin and increasing the resistance of the formed clot against fibrinolysis [114]. Notably, patients with “unexplained” intraoperative coagulopathies and, hence, bleeding demonstrate significantly less FXIII per unit thrombin available both before, during and after surgery [115]. An increased tendency to postoperative bleeding has been observed, even at factor XIII activities as high as 60% [116]. The role of FXIII administration to bleeding trauma patients however remains to be investigated in randomized clinical trials.

### Teragnostic approach

Recently, a new approach to resuscitation of patients with massive blood loss was introduced, employing goal-directed administration of pharmacological agents such as fibrinogen concentrate, prothrombin complex concentrate, recombinant factor VIIa and factor XIII as alternatives to FFP and platelet concentrates together with volume resuscitation with synthetic colloids. Observational studies of limited size of such an approach in injured patients with massive blood loss indicate that it may be feasible to achieve hemostasis with the use of these procoagulant agents [117].

It should be noted, however, and especially in the light of the disappointing results from the trial regarding the use of recombinant factor VII in trauma, that adequately powered double-blind randomized studies are required before routine use of goal-directed administration of procoagulant agents to injured patients with bleeding is recommended. This is especially important with regard to the safety of these agents, which, as opposed to FFP, do not contain relevant concentrations of natural anticoagulant factors such as antithrombin, protein C and protein S, and this may be of importance in the later phase after hemostatic control has been established [118].

Additionally, it remains elusive which fluid that should be used to support the hemodynamic system during massive bleeding treated with the teragnostic approach when plasma not is included in the resuscitation regimen. Crystalloids clearly have not sufficient volume expanding effects and based on the findings of increased bleeding, transfusion requirements and mortality in septic patients receiving synthetic colloids [119] it could be feared that similar results would surfer in severely injured trauma patients.

## Conclusions

Viscoelastic whole blood assays, such as TEG and ROTEM are advantageous for identifying coagulopathy, and guide ongoing transfusion therapy. From the result of these assays, implementation of a hemostatic control resuscitation strategy to massively bleeding patients seems both reasonable and lifesaving although data from prospective randomized controlled trials are lacking. Until definite proof from such trials is available, retrospective data support a shift in transfusion medicine in regard to early and aggressive administration of plasma and platelets.

## Competing interest

PJO has received support with analyzers and reagents from Hemonetic Corp, TEM International and Viber Int. PJO has received unrestricted research grants from Novo Nordisk AS and Octaparma GmbH. JS and SRO declare no conflicts of interest.

## Author contribution

PJ, JS and SRO contributed to the conception and design of the manuscript, literature search, and writing of the manuscript. All authors read and approved the final manuscript.
